# Optic flow parsing in the macaque monkey

**DOI:** 10.1167/jov.20.10.8

**Published:** 2020-10-05

**Authors:** Nicole E. Peltier, Dora E. Angelaki, Gregory C. DeAngelis

**Affiliations:** 1 Department of Brain and Cognitive Sciences, Center for Visual Science, University of Rochester, Rochester, NY, USA; 2 Center for Neural Science, New York University, New York, NY, USA; 3 Department of Brain and Cognitive Sciences, Center for Visual Science, University of Rochester, Rochester, NY, USA

**Keywords:** optic flow, motion perception, monkey, self-motion

## Abstract

During self-motion, an independently moving object generates retinal motion that is the vector sum of its world-relative motion and the optic flow caused by the observer's self-motion. A hypothesized mechanism for the computation of an object's world-relative motion is flow parsing, in which the optic flow field due to self-motion is globally subtracted from the retinal flow field. This subtraction generates a bias in perceived object direction (in retinal coordinates) away from the optic flow vector at the object's location. Despite psychophysical evidence for flow parsing in humans, the neural mechanisms underlying the process are unknown. To build the framework for investigation of the neural basis of flow parsing, we trained macaque monkeys to discriminate the direction of a moving object in the presence of optic flow simulating self-motion. Like humans, monkeys showed biases in object direction perception consistent with subtraction of background optic flow attributable to self-motion. The size of perceptual biases generally depended on the magnitude of the expected optic flow vector at the location of the object, which was contingent on object position and self-motion velocity. There was a modest effect of an object's depth on flow-parsing biases, which reached significance in only one of two subjects. Adding vestibular self-motion signals to optic flow facilitated flow parsing, increasing biases in direction perception. Our findings indicate that monkeys exhibit perceptual hallmarks of flow parsing, setting the stage for the examination of the neural mechanisms underlying this phenomenon.

## Introduction

As we move through the world, our self-motion generates a structured pattern of optic flow on our retinas (Gibson, 1950; Longuet-Higgins & Prazdny, 1980). Optic flow carries information about one's instantaneous direction of translation, or heading, as well as information about eye rotation relative to the scene (Koenderink, 1986; Koenderink & van Doorn, 1987; Longuet-Higgins & Prazdny, 1980; Prazdny, 1983). Extensive research has demonstrated that humans can use optic flow to discriminate their heading (e.g., Foulkes, Rushton, & Warren, 2013b; Van den Berg, 1992; W. H. Warren, Blackwell, Kurtz, Hatsopoulos, & Kalish, 1991; W. H. Warren & Hannon, 1988), even in the presence of moving objects, which cause small, systematic biases under some conditions (Li, Ni, Lappe, Niehorster, & Sun, 2018; Royden & Hildreth, 1994, 1996; W. H. Warren & Saunders, 1995). Often, however, we must be able to interpret an object's motion while we are also moving. When running to catch a baseball or driving down a busy city street, it can be important to estimate how an object is moving relative to the world, rather than relative to our moving selves. To do this, we must compensate for our self-motion to transform the object's motion from retinal coordinates to world-centered coordinates.

An object's motion in retinal coordinates is the vector sum of its motion in world coordinates and the optic flow produced by the observer's self-motion. A proposed mechanism for the computation of an object's world-relative motion is flow parsing, in which the visual system subtracts the optic flow due to self-motion from the object's retinal image motion (P. A. Warren & Rushton, 2007, 2008, 2009a, 2009b). As a result of this subtraction, the perception of an object's motion will be, relative to its retinal motion, biased away from the direction of optic flow at the location of the object (Dokka, MacNeilage, DeAngelis, & Angelaki, 2015; Fajen & Matthis, 2013; Fajen, Parade, & Matthis, 2013; Foulkes, Rushton, & Warren, 2013a; Rogers, Rushton, & Warren, 2017; Rushton & Warren, 2005; P. A. Warren & Rushton, 2007, 2008, 2009a, 2009b). Perceptual hallmarks of flow parsing are robust to sparse optic flow density (Foulkes et al., 2013a; Royden & Connors, 2010), noisy optic flow (Foulkes et al., 2013a), and the removal of stereo depth cues from the optic flow field (P. A. Warren & Rushton, 2009a). Biases in perceived object direction due to optic flow persist even when the flow field is masked in the entire visual hemifield surrounding the probe object (P. A. Warren & Rushton, 2009b), suggesting that there is a global component to flow parsing and that the visual system does not rely solely on a local comparison of an object's retinal motion with nearby optic flow vectors.

**Figure 1.**
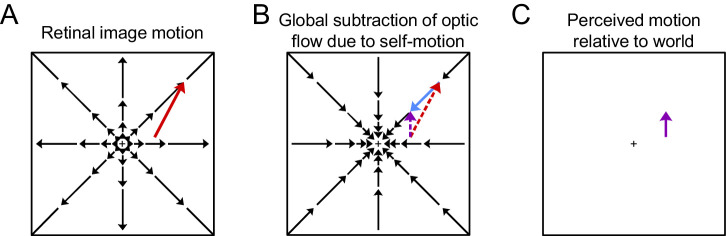
The flow-parsing hypothesis explains how the visual system might compute an object's motion during self-motion. (A) During forward self-motion, optic flow expands radially outward from a central focus of expansion. The retinal motion of an independently moving object will be the vector sum of its motion relative to the world and the optic flow vector at its location. In this illustration, an object moving upward in the right visual field will have a rightward component added to its motion (red). (B) To compute the object's motion relative to the world, the visual system globally subtracts the optic flow that resulted from self-motion. A leftward component (blue) is added to the independently moving object's motion. (C) After parsing out the optic flow, any remaining motion is due to the motion of the object relative to the world (purple).

Figure 1 illustrates the flow-parsing process. During forward self-motion, optic flow vectors expand radially on the retina from a central focus of expansion. An independently moving object will produce retinal motion that is a combination of its world-relative motion with the radial optic flow vector at its location (Figure 1A). To compute the object's motion relative to the world, the flow-parsing hypothesis purports that the visual system globally subtracts off the optic flow field resulting from self-motion. In the case of forward self-motion, this is equivalent to a radial contraction field being added to velocity vector field at each point in the image (Figure 1B). The result of this computation is an estimate of the object's motion relative to the world (Figure 1C). Thus, the flow-parsing hypothesis predicts that the observer's perceived direction of object motion should be biased away from the optic flow vectors produced by self-motion in the vicinity of the object. Note that biases induced by flow parsing are typically described in display screen (or retinal) coordinates. If flow parsing occurs perfectly, then an unbiased estimate of object motion in world coordinates corresponds to a bias in screen coordinates.

There is extensive psychophysical evidence in favor of the flow-parsing hypothesis (Dupin & Wexler, 2013; Foulkes et al., 2013a; Rushton, Bradshaw, & Warren, 2007; Rushton & Warren, 2005; P. A. Warren & Rushton, 2007, 2008, 2009a, 2009b; P. A. Warren, Rushton, & Foulkes, 2012), but we know very little about the neural mechanisms underlying this process. While some recent human neuroimaging studies have reported neural correlates of flow parsing (Field, Biagi, & Inman, 2020; Pitzalis et al., 2020), nothing is currently known about how flow parsing is implemented at the level of individual neurons or neural populations. To investigate the neurophysiology of flow parsing, we must first develop an animal model of flow-parsing behavior. In these experiments, we sought to elucidate whether rhesus monkeys exhibit flow-parsing behavior that follows the same patterns described in human psychophysics. Once we establish that monkeys show the same types of biases in object motion perception due to the presence of optic flow, we can investigate the neural substrates that represent this process.

**Figure 2.**
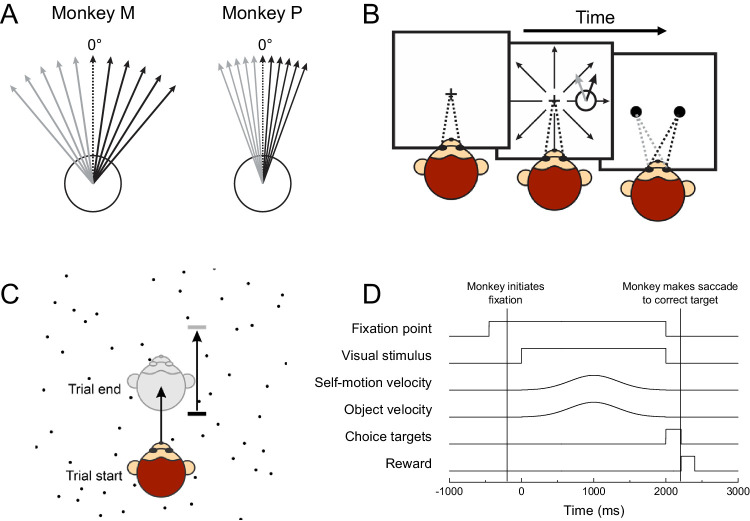
Schematic illustration of the object direction discrimination task. (A) The object moved in 1 of 11 linearly spaced directions centered on straight upward. Monkey M discriminated object direction within a range of ± 40°, while Monkey P discriminated object direction within a range of ± 20°. 0° denotes object motion straight upward (in screen coordinates). (B) Each trial initiated when a fixation target appeared and the monkey fixated on the target. The monkey was required to maintain fixation during the presentation of a stimulus, which consisted of an object moving upward obliquely and a global optic flow field simulating forward or backward self-motion. At the end of the stimulus presentation, two choice targets appeared. The monkey was required to make a saccade to one of the targets indicating whether the object's motion was rightward or leftward of vertical. (C) Top-down schematic illustrating motion of subject and object in depth over the course of the trial. Optic flow simulated the monkey's self-motion through a stationary cloud of dots. The object moved in depth relative to the world so that it stayed at a constant distance from the monkey. (D) Timeline of events within each trial.

Because the pattern of an optic flow field is determined mathematically (Longuet-Higgins & Prazdny, 1980), one can predict the size of the perceptual bias induced by flow parsing for each location in the visual field. We tested how well the flow-parsing hypothesis predicted perceptual biases under different self-motion conditions and scene arrangements. Our experiments employed a two-alternative forced-choice (2AFC) task in which subjects discriminated whether an object moved to the right or left of vertical in the presence of optic flow (Figure 2). Because the task involved discriminating the direction of the horizontal component of object motion, only the horizontal component of background optic flow was pertinent to generating perceptual biases. We used optic flow stimuli that simulated forward and backward self-motion, for which the horizontal component of an optic flow vector, *u*, is given by (Longuet-Higgins & Prazdny, 1980):

(1) $$u=\frac{WX}{Z^{2}},$$

where *W* denotes forward or backward self-motion velocity, *X* represents the horizontal location of the optic flow vector relative to the observer, and *Z* denotes the depth of the optic flow vector relative to the observer.

In our 2AFC task, a subject that performs flow parsing may report the object's motion to be in one direction (e.g., rightward) when its motion on the display is in the opposite direction (e.g., leftward). This type of reversal would occur when *u*, computed at the location of the object, is in the same direction and of greater magnitude as the horizontal component of the object's retinal motion, such that subtraction of *u* reverses the direction of the horizontal component. Therefore, factors that affect an optic flow vector's horizontal velocity—*W*, *X*, and *Z*—should affect whether retinal and world-centered coordinates disagree.

Self-motion velocity, *W*, is directly related to an optic flow vector's magnitude (Equation 1). As we move through the world faster, the world passes by our retinas more quickly. When optic flow vectors are longer, subtraction of these vectors from an object's retinal motion will yield more substantial biases in object motion perception. If the magnitude of an optic flow vector is large enough, this subtraction may reverse the subject's report regarding whether object direction is rightward or leftward of vertical. During forward self-motion, this could happen when an object in the right visual field is moving rightward relative to the world or when an object in the left visual field is moving leftward relative to the world. Backward self-motion, indicated by negative values of *W*, is expected to bias perception in the opposite direction, such that a subject's report of object direction may reverse for a leftward-moving object in the right visual field or a rightward-moving object in the left visual field. Faster speeds of self-motion should make it more likely that the percept of object direction is reversed relative to the object's retinal motion.

The object's horizontal location, *X*, is also directly related to the magnitude of an optic flow vector's horizontal component at that location (Equation 1). During forward and backward self-motion, peripheral optic flow vectors have greater length (speed) than central flow vectors, such that the horizontal component speed of flow vectors increases for larger horizontal eccentricities. Thus, we expect perceptual biases regarding object direction to grow with horizontal eccentricity of the object's location, whereas biases should be largely independent of vertical eccentricity.

The horizontal component of optic flow also decreases with the square of the flow vector's distance from the observer, *Z*^2^ (Equation 1), such that far optic flow vectors are of smaller magnitude than near ones. Subtraction of these flow vectors is expected to have a smaller effect on the perception of object direction when the object is located at a farther distance. Therefore, we expect flow parsing to produce smaller perceptual biases for far than near objects (that have the same retinal velocity).

Most studies of flow parsing have not determined whether optic flow is sufficient for people to compensate entirely for their self-motion, and those that have done so have generally reported biases smaller than those predicted by flow parsing (Dokka et al., 2015; Fajen et al., 2013; Layton & Niehorster, 2019; Niehorster & Li, 2017). Incomplete or imperfect flow parsing may be attributed to the fact that optic flow is not the only sensory consequence of self-motion. Vestibular signals can also be used to discriminate heading in both humans (Benson, Spencer, & Stott, 1986; Jones & Young, 1978; MacNeilage, Banks, DeAngelis, & Angelaki, 2010) and monkeys (Gu, DeAngelis, & Angelaki, 2007), and the addition of vestibular self-motion cues to optic flow reduces variance in heading perception (Butler, Smith, Campos, & Bulthoff, 2010; Fetsch, Turner, DeAngelis, & Angelaki, 2009; Gu, Angelaki, & DeAngelis, 2008). While others have demonstrated that vestibular signals can influence the perception of object motion during self-motion (Dokka et al., 2015; Fajen & Matthis, 2013; Hogendoorn, Verstraten, MacDougall, & Alais, 2017; MacNeilage, Zhang, DeAngelis, & Angelaki, 2012), no one has directly tested whether vestibular self-motion cues accentuate the biases in perceived object direction that are expected from flow parsing. We incorporated vestibular self-motion cues to investigate whether a multisensory estimate of self-motion leads to more complete compensation for self-motion.

## General methods

### Subjects and materials

Two male monkeys (*Macaca mulatta*) participated in these experiments. A head restraint device was implanted according to standard aseptic surgical procedures under gas anesthesia. Specifically, a Delrin (Dupont, Wilmington, DE) ring was attached to the skull with dental acrylic cement and anchored with bone screws and titanium inverted T-bolts (see Gu, Watkins, Angelaki, & DeAngelis, 2006, for details). To monitor eye movements, a scleral coil was implanted under the conjunctiva of one eye. All surgical procedures and experimental protocols were approved by the University Committee on Animal Resources at the University of Rochester.

Monkeys were seated in custom-made primate chairs, which were mounted on a 6-degree-of-freedom motion platform (Moog 6DOF2000E, Elma, NY). This platform remained stationary in all experiments except Experiment 4. A field coil frame (C-N-C Engineering, Seattle WA) was mounted on the motion platform to monitor eye movements using a scleral search coil technique.

Visual stimuli were rear-projected onto a 60-cm × 60-cm tangent screen using a stereoscopic projector (Christie Digital Systems, Cypress CA, Mirage S+3K) that was mounted on the motion platform. The display screen was attached to the front of the field coil frame, approximately 30 cm in front of the animal (Monkey M: 31.7 cm from eyes to screen; Monkey P: 33.0 cm from eyes to screen). As a result, the screen subtended approximately 90 × 90° of visual angle. The sides and top of the field coil frame were covered with black matte cardboard to restrict the monkey's field of view to the visual stimuli on the screen.

### Visual stimuli

**Figure 3.**
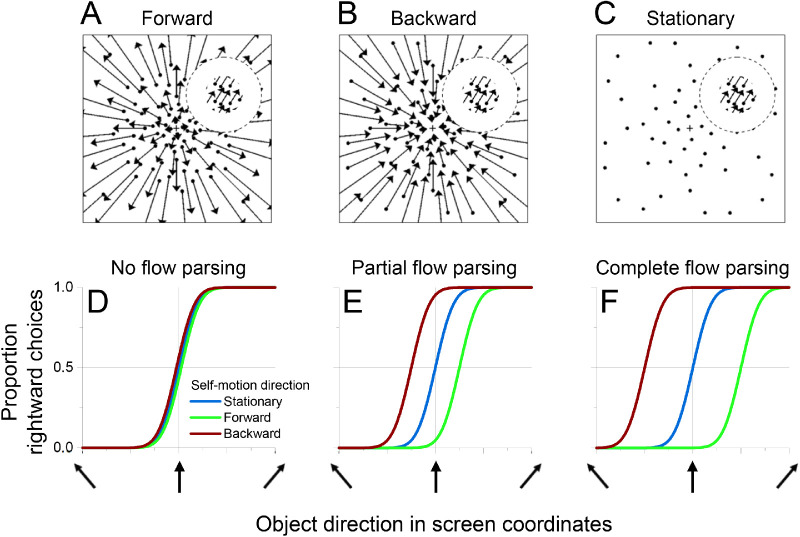
Schematic illustration of visual stimuli and psychometric functions. (A–C) Illustration of visual stimuli under different self-motion conditions. The small patch of dots, which represents the object to be discriminated, moves to the right or left of straight upward. A circular mask surrounds the object to prevent local motion comparisons between the object and the background optic flow. (A) In the forward self-motion condition, optic flow expands radially outward from a central focus of expansion. (B) In the backward condition, optic flow contracts radially toward a central focus of contraction. (C) In the stationary condition, background dots were static. Note that the motion of the target object on the display is identical for each of the background motion conditions. (D–F) Schematic illustrations of psychometric functions illustrating different amounts of flow parsing. Functions illustrate the proportion of rightward judgments the subject made as a function of object direction in screen-relative coordinates. Line color indicates self-motion condition (blue: stationary, green: forward, red: backward). (D) If the subject does not flow parse at all, psychometric curves will lie on top of each other. (E) If the subject performs partial flow parsing, psychometric curves for forward and backward conditions will shift horizontally. (F) If the subject performs complete flow parsing, indicating object motion in world-centered coordinates, psychometric curves for forward and backward conditions will be substantially separated. The size of the shift predicted by flow parsing depends on self-motion speed and object location.

The visual stimulus simulated the motion of an independently moving object during forward or backward self-motion (Figure 3). Some aspects of the stimuli differed between experiments; these differences will be detailed when each experiment is discussed below. Stimuli were generated by software written in Visual C++, using the OpenGL (Silicon Graphics Inc., Mountain View, CA) 3D graphics rendering library. An OpenGL camera located at the same position as each of the animal's eyes generated the planar image projection shown to each eye. To simulate depth in the scene, stimuli were rendered stereoscopically as red/green anaglyphs, and they were viewed by the animal through red and green filters (Kodak, Rochester NY, Wratten2 #29 and #61, respectively). Visual stimuli were presented for 2,000 ms while the animal maintained visual fixation.

#### Object motion

Object motion was represented by random dots moving coherently within a circular aperture. The diameter of the aperture varied slightly across experiments but was typically around 10 cm, which corresponds to 17.5° for Monkey M and 16.9° for Monkey P. Slight variations in retinal object diameter between animals are due to small differences in viewing distance. During a trial, dots would reach the edge of the aperture and then be extinguished as new dots would appear at the opposite edge of the aperture. This phenomenon is equivalent to a large object moving behind an aperture. Direction and speed of the object's motion refer to the direction and speed of the dots within the object or, equivalently, to the direction and speed of a large object moving behind the aperture. Dots were distributed within a nearly flat (frontoparallel) ellipsoid with a depth range of 0.1 cm. Dots within the object were rendered as triangles 0.15 cm wide and 0.15 cm tall, and they were distributed with a density of 20 dots/cm^3^.

Apertured motion was used so that the object motion remained within a fixed retinal location, preventing subjects from basing their direction discrimination responses on positional cues. The stationary aperture was also advantageous for neural recording experiments (to be described elsewhere), as the object remained centered on a neuron's visual receptive field for the duration of the trial. For one animal, we also performed behavioral experiments in which the boundaries of the object moved together with the dots within, as is naturally the case for real objects. We observed no qualitative differences in discrimination thresholds or in biases induced by optic flow; thus, flow parsing appears to function for both “objects” with stationary or moving boundaries.

The object was positioned in opposite visual hemifields for the two subjects because they had different hemispheres prepared for neural recordings (to be described elsewhere). For the monkey with its right hemisphere prepared for recording (Monkey M), the object was placed on the left side of the visual field. For the monkey with its left hemisphere prepared (Monkey P), the object was placed on the right side of the visual field. The precise location and size of the object were determined independently for each experiment, as described below.

The object moved within the frontoparallel plane in 1 of 11 directions centered on straight upward. We used a substantially wider range of object directions than is necessary to measure direction discrimination thresholds without background motion (e.g., Purushothaman & Bradley, 2005). This was because optic flow was expected to produce substantial biases in subjects’ psychometric functions, and a wider range of object directions allowed for the measurement of these biases. For one monkey (Monkey P), object directions ranged from –20° to +20° relative to straight upward, linearly spaced in increments of 4° (Figure 2A). For the other monkey (Monkey M), object directions ranged from –40° to +40 in increments of 8°, since this animal showed substantially larger biases associated with flow parsing. Linear spacing of object directions allowed us to measure biases with equal resolution across the range of tested directions. The 11 distinct object directions were interleaved randomly within each block of trials. Dots within the object moved coherently following a Gaussian velocity profile with a standard deviation, σ, of 0.33 s, hitting peak speed in the middle of the trial. The precise peak speed of the object was determined independently for each experiment, as described below.

During trials with self-motion, object motion on the display was consistent with that generated by an object that moved in depth with the observer, thus keeping the object's position and size constant on the retina (Figure 2C). The direction and speed of object motion were therefore defined in a frontoparallel plane that moved forward or backward with the simulated self-motion. Thus, object motion on the display (and in turn on the retina, ignoring any fixation error) was identical for a given object direction under different optic flow conditions. Thus, any observed differences in perceived object direction between self-motion conditions could be attributed to the effect of surrounding optic flow.

#### Self-motion

Surrounding the object was a three-dimensional cloud of background dots extending in depth from 5 cm to 55 cm from the eyes. This cloud consisted of triangles 0.1 cm tall and 0.1 cm wide, distributed with a density of 0.002 dots/cm^3^. The retinal size of the background dots varied inversely with distance from the observer, adding a monocular depth cue to the optic flow and simulating self-motion more realistically.

In most trials, the OpenGL cameras moved through the cloud of dots, generating an optic flow pattern that simulated self-motion. The dots expanded radially to simulate forward self-motion (Figure 3A), and they contracted to simulate backward self-motion (Figure 3B). In a set of control trials, the background dots remained static to indicate no self-motion (Figure 3C). Self-motion followed the same Gaussian velocity profile as object motion with σ = 0.33 s, hitting peak self-motion speed in the middle of the trial. Because self-motion speeds were not constant throughout a trial, we will refer to the magnitude of self-motion as its amplitude, or the total distance traveled by the subject throughout the trial. At least two different self-motion amplitudes were used in each experiment. Self-motion conditions (forward, backward, stationary) and amplitudes were all interleaved within blocks of trials.

A portion of the optic flow field directly surrounding the object was masked to prevent subjects’ responses from being driven strongly by local motion interactions. This mask is also beneficial for neural recordings (to be described elsewhere), as it keeps optic flow outside of receptive fields and nonclassical surrounds. The size of the mask was determined by a mask ratio, which is the ratio of the mask's diameter to the object's diameter. The mask ratio was 2 for all experiments except Experiment 2, for which the mask ratio was 1.5.

### Procedure

On each trial, a fixation point appeared in the center of a blank screen. The subject initiated the trial by moving its eyes to the fixation point. The subject's eyes had to remain fixated within a 2.5° to 2.8° (full width) box surrounding the fixation point, or the trial would abort. After a 200-ms delay, the visual stimulus was presented. The stimulus, which lasted for 2 s, showed simultaneous object motion and optic flow. Immediately after the stimulus ended, the screen went blank and two choice targets appeared, 5° on either side of the fixation point. The subjects indicated whether they perceived rightward or leftward object motion by making a saccade to the respective choice target. Correct responses were rewarded with a drop of juice (∼0.2 ml on average).

For most trials, the correct answer was the same regardless of whether the subject perceived object motion in retinal or world coordinates. On those unambiguous trials, subjects were rewarded on 95% of correct trials. Trials for which flow parsing might reverse the perceived direction of the object were deemed ambiguous, and 70% of these trials were rewarded regardless of the subject's response. This reward paradigm was used so as not to encourage subjects to use one coordinate frame over the other.

### Analysis

#### Psychometric analysis

For each experimental session, we computed a psychometric function, for each distinct self-motion and object location condition, that describes the proportion of rightward choices as a function of object direction. We calculated the probability of a rightward choice, given the object's direction in screen coordinates, as a cumulative Gaussian distribution, given by

(2) $$\;P\left(rightward\;choice\;|\;\theta \right)=\;\frac{1}{2}\left(1+\mathrm{erf}\left(\frac{\theta -\mu }{\sigma \sqrt{2}}\right)\right),$$

where *θ* denotes the object's direction of motion in screen coordinates; *µ* represents the point of subjective equality (PSE) or bias; *σ* denotes the distribution's standard deviation, which is taken as the psychometric threshold; and erf(*x*) represents the Gauss error function given by

(3) $$\mathrm{erf}\left(x\right)=\frac{2}{\sqrt{\pi }}\int_{0}^{x}e^{-t^{2}}dt.$$

Parameters *µ* and *σ* were optimized to minimize the sum squared error between the predicted proportion of rightward choices and the measured proportion of rightward choices. A lapse rate parameter was not included because the subjects’ psychometric functions generally did not reveal substantive lapses in the control condition with stationary background dots.

The mean of the psychometric function, *µ*, represents the direction of object motion on the screen at which the monkey makes 50% rightward choices and 50% leftward choices. Since psychometric functions are always plotted as a function of object direction in screen coordinates, flow parsing is expected to induce biases in perceived object direction and corresponding shifts of the PSE (Figure 2D–F). If the monkey does not flow parse at all, but rather just reports object motion in retinal coordinates, then we would not expect any shifts in the measured psychometric functions. The effect of optic flow on perceived object direction was measured as the difference in PSE between forward and backward optic flow conditions:

(4) $$\begin{array}{ccc} \;PSE\;shift & = & \;\mathrm{sign}\left(ObjXLocation\right)\;\\ & & \times (PSE_{forward}-PSE_{backward}),\; \end{array}$$

where sign(*ObjXLocation*) is +1 for objects located in the right visual field and –1 for objects located in the left visual field, *PSE_forward_* is the PSE of the psychometric function for the forward optic flow condition, and *PSE_backward_* is the PSE for backward optic flow. The expected effect of flow parsing depends on the horizontal direction of optic flow vectors implied at the location of the object, which depends on the object's location. As a result, PSE shifts incorporate the sign of the object's horizontal position such that values are always positive if they are in the direction predicted by flow parsing. In Experiment 2, when the object is on the vertical meridian (zero horizontal position) such that its sign is undefined, we applied the same multiplier as used for the other object locations that were tested in the same session. This allowed us to see if any perceptual biases observed for the object on the vertical meridian are in the same direction as the effects at the other object locations.

#### Computing PSE shift predicted by flow parsing

We predicted the size of the perceptual bias induced by flow parsing using equations that describe the optic flow field under planar image projection (Longuet-Higgins & Prazdny, 1980). A point with instantaneous coordinates (*X*, *Y*, *Z*) relative to an observer changes position relative to the observer with velocity components given by

(5) $$\begin{array}{c} \dot{X}=-U-BZ+CY\\ \dot{Y}=-V-CX+AZ\\ \dot{Z}=-W-AY+BX \end{array}$$

where *A*, *B*, and *C* represent the observer's rotational velocities along the pitch, yaw, and roll axes, respectively, and *U*, *V*, and *W* denote the observer's translational velocities in the lateral, heave, and surge directions, respectively.

This point's instantaneous coordinates are projected into two-dimensional image coordinates as follows:

(6) $$\left(x,y\right)=\left(\frac{X}{Z},\frac{Y}{Z}\right).$$

During self-motion, this point will move across the image with velocity

(7) $$\left(u,v\right)=\left(\dot{x},\dot{y}\right).$$

Substitution from Equations 5 and 6 into Equation 7 yields

(8) $$\begin{array}{ccc} u & = & \frac{\dot{X}}{Z}-\frac{X\dot{Z}}{Z^{2}}=\left(-\frac{U}{Z}-B+Cy\right)\\ & & -x\left(-\frac{W}{Z}-Ay+Bx\right), \end{array}$$

(9) $$\begin{array}{ccc} v & = & \frac{\dot{Y}}{Z}-\frac{Y\dot{Z}}{Z^{2}}=\left(-\frac{V}{Z}-Cx+A\right)\\ & & -y\left(-\frac{W}{Z}-Ay+Bx\right). \end{array}$$

To convert the retinal image projection of an object's motion to world coordinates, one must subtract *u* and *v* for a point at the object's (*X*, *Y*, *Z*) location.

Our experiments simulated forward and backward self-motion without rotations, so translational components *U* and *V*, as well as rotational components *A*, *B*, and *C*, were zero. Equations 8 and 9 can therefore be simplified as follows:

(10) $$u=\frac{Wx}{Z}.$$

(11) $$v=\frac{Wy}{Z}.$$

Substitution of Equation 6 into Equation 10 generates the representation of an optic flow vector's horizontal component of retinal velocity as seen in Equation 1. Substitution of Equation 6 into Equation 11 represents the vertical component of an optic flow vector's retinal velocity, shown as follows:

(12) $$v=\frac{WY}{Z^{2}}.$$

The bias due to flow parsing can be predicted as a function of the object's location and velocity, as well as the observer's self-motion velocity using the equations described above. We computed the expected bias for each self-motion condition as the object direction on the screen that would be perceived as moving straight upward if subjects were to flow parse completely. At this critical object direction, the object's horizontal component is identical in direction and magnitude to the horizontal component of optic flow at the object's location. An object moving in a direction between the critical direction and straight upward would have a horizontal component smaller than that of the coincident optic flow vector, such that subtraction of the optic flow would bias perception from one side to the other.

Predicted biases due to flow parsing were computed from the amplitudes of self-motion and object motion. However, because the Gaussian velocity profiles were the same between object motion and optic flow, there was a constant relationship between the two velocities throughout the trial. As a result, expected biases due to flow parsing would be consistent regardless of whether amplitudes, mean velocities, or velocities at any point of the trial were used in the calculations.

#### Flow-parsing gains

We compared the measured perceptual biases with those predicted from flow parsing by computing a *flow-parsing gain* (Layton & Niehorster, 2019; Niehorster & Li, 2017). The flow-parsing gain is the ratio of the observed PSE shift to the PSE shift that is predicted by completely subtracting out the optic flow:

(13) $$\text{Flow-parsing}\;\text{gain}=\frac{PSE\;shift_{observed}}{PSE\;shift_{predicted}}.$$

Flow-parsing gain will be 1 if the subject completely parses out optic flow and reports the object's motion in world coordinates, and it will be 0 if the subject does not parse out optic flow at all, indicating the object's motion in retinal coordinates.

#### Statistics

PSE shifts and flow-parsing gains were compared across conditions using linear regression (function fitlm in MATLAB). In cases of more than one predictor variable, multiple regression was used, including interactions between predictor variables. Prior to each regression, each numerical variable was mean-centered by subtracting the mean from all observations of that variable. Separate regressions were calculated for each monkey.

## Experiment 1: Effect of self-motion amplitude

In this experiment, two monkeys performed the discrimination task described above while optic flow simulated self-motion directly forward or backward (Figure 2). We manipulated both the direction and amplitude of simulated self-motion to examine how closely object motion perception during self-motion matches the predictions of flow parsing. We tested both forward and backward self-motion directions because the flow-parsing hypothesis predicts opposite perceptual biases for these two patterns of optic flow.

The rate of optic flow field expansion and contraction was manipulated to simulate three different speeds of self-motion. Because self-motion followed a Gaussian velocity profile, we varied the amplitude of self-motion (total displacement during a trial), which scales proportionally with self-motion speed. Faster self-motion produces longer optic flow vectors, so the flow-parsing hypothesis predicts greater biases in perceived object direction for greater amplitudes of self-motion.

### Methods

Two monkeys participated in this experiment. The object was placed in the fixation plane at a location where each monkey had previously been trained extensively to perform direction discrimination. For Monkey M, the object was placed 11 cm (19.2°) to the left of the fixation point on the horizontal meridian. For Monkey P, the object was placed 14.7 cm (23.6°) to the right of the fixation point. The object's diameter was approximately two thirds of its eccentricity (7.73 cm for Monkey M, 9.38 cm for Monkey P), and the background mask surrounding the object had a diameter that was twice the object's diameter. The object's motion amplitude was set such that it hit a peak speed of 10°/s in the middle of the trial. Over the course of the trial, the dots within the object traveled 6.68° on the screen (3.69 cm of screen-relative motion for Monkey M and 3.92 cm for Monkey P).

Optic flow simulated forward or backward self-motion at one of three speeds. The amplitudes of self-motion were determined in order to produce specific expected PSE shifts according to flow parsing. The expected PSE shifts, assuming complete flow parsing, were 4°, 8°, and 16° for Monkey M and 6°, 20°, and 40° for Monkey P. These disparate expected PSE shifts were chosen because preliminary testing revealed that the gain of flow parsing was much greater for Monkey M than for Monkey P, as documented below. Thus, choosing different self-motion amplitudes for the two animals made the shifts readily measurable in both animals.

There were 11 unique object directions and 7 self-motion conditions (three forward speeds, three backward speeds, and a stationary background condition), for a total of 77 unique stimulus conditions. Monkey M completed 6,930 trials over six sessions (one session per day), and Monkey P completed 5,313 trials over four sessions. In each session, monkeys worked to satiety; thus, the number of trials completed was not identical across sessions. Monkey M completed 14 to 16 repetitions of each unique stimulus per session, whereas Monkey P completed 14 to 20 repetitions.

### Results

**Figure 4.**
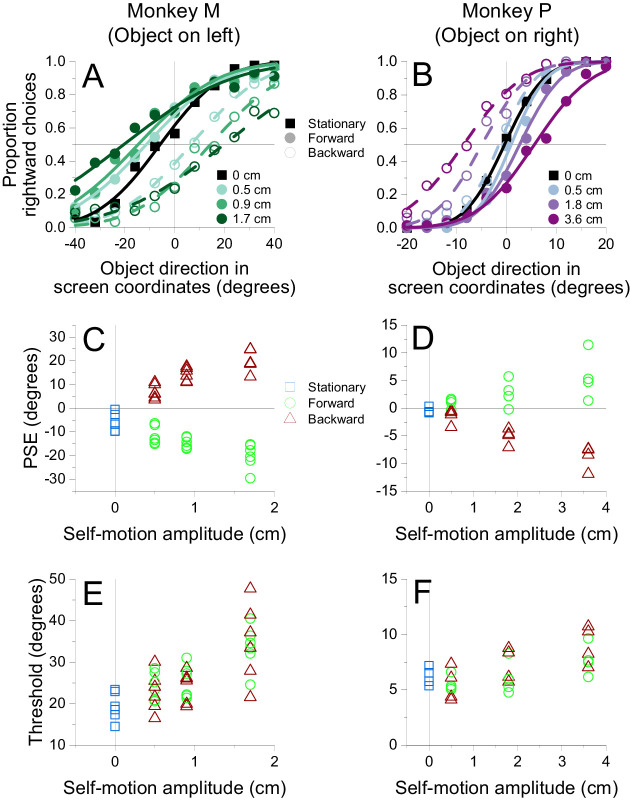
Perceived object motion is biased depending on the direction and speed of optic flow. (A, B) Summary psychometric curves from monkeys discriminating object direction in the presence of optic flow (6,930 trials from Monkey M; 5,313 trials from Monkey P). Smooth curves show fits of a cumulative Gaussian function to the data points. Symbols denote data from the stationary (squares), forward (filled circles), and backward (open circles) self-motion conditions. (A) For Monkey M, when the object was in the left visual field, perceived direction was biased rightward during forward self-motion and leftward during backward self-motion. The amount of bias increased with the amplitude of self-motion. (B) For Monkey P, when the object was in the right visual field, perceived direction was biased leftward during forward self-motion and rightward during backward self-motion. (C) PSEs for Monkey M are plotted as a function of self-motion amplitude. Colors denote the self-motion condition: stationary (blue), forward (green), and backward (red). Multiple points for each self-motion amplitude indicate results from individual sessions. (D) PSEs for Monkey P are plotted against self-motion amplitude; format as in panel C. (E, F) Psychophysical thresholds are plotted as a function of self-motion amplitude for Monkeys M and P, respectively. Format as in Panels C and D.

Data were combined across sessions for each animal and were compiled into psychometric functions that describe the proportion of rightward choices as a function of object direction in screen coordinates (Figure 4A and B). Forward and backward self-motion clearly induced opposite biases in object motion perception. Because the retinal image motion of the object was identical across self-motion conditions, these biases must be driven by the surrounding optic flow. When the object is in the left visual field (Figure 4A, Monkey M), perception is biased rightward during forward self-motion and leftward during backward self-motion. The directions of these biases are as predicted by flow parsing. During forward self-motion, optic flow vectors at the location of the object (left hemifield) are moving leftward, such that subtraction of these flow vectors should cause a rightward bias in perceived object direction. The converse is true for backward self-motion. When the object is located in the right visual hemifield (Figure 4B, Monkey P), the effect of optic flow on perceptual biases is reversed, as expected from the flow-parsing hypothesis: Perception is biased leftward during forward self-motion and rightward during backward self-motion. This reversal of bias is due to the symmetry of the optic flow field around straight ahead.

For both object locations, the horizontal separation between psychometric curves corresponding to forward and backward self-motion increases with the amplitude of self-motion (Figure 4A and B). This effect is also consistent with flow parsing. As self-motion amplitude increases, subtraction of longer optic flow vectors at the location of the object should induce a greater perceptual bias.

To quantify the biases induced by optic flow, we fit each psychometric curve with a cumulative Gaussian function. The object direction at which the curve crosses 50% rightward choices is the PSE, or the object direction at which the monkey makes an equal number of rightward and leftward reports. We computed a separate PSE for each self-motion condition of each session.

PSEs are plotted as a function of self-motion amplitude in Figure 4C and D. Both plots show a linearly increasing separation between PSE values corresponding to forward and backward self-motion as self-motion amplitude increases, reflecting larger perceptual biases induced by faster optic flow. Multiple linear regressions were conducted to test for the effect of self-motion direction, self-motion amplitude, and the interaction between the two variables on PSE. Because the stationary condition does not have a direction, only forward and backward self-motion conditions were included in these regressions. The regressions accounted well for the data in both subjects (Monkey M: *R*^2^ = 0.9477, *F*(3, 32) = 193.2, *p* = 1.425 × 10^−20^; Monkey P: *R*^2^ = 0.8495, *F*(3, 20) = 37.62, *p* = 2.056 × 10^−8^). There was a main effect of self-motion direction in both animals (Monkey M: β = –26.58, *t*(32) = –20.15, *p* = 9.619 × 10^−20^; Monkey P: β = –7.159, *t*(20) = –7.6713, *p* = 2.215 × 10^−7^), reflecting biases in opposite directions that are induced by forward and backward self-motion. There was also a significant main effect of self-motion amplitude (Monkey M: β = 711.3, *t*(32) = 3.966, *p* = 3.853 × 10^−4^; Monkey P: β = 154.5, *t*(20) = 3.048, *p* = 6.354 × 10^−3^), which indicates greater biases as the magnitude of optic flow increased. There was a significant interaction between self-motion direction and self-motion amplitude for both subjects (Monkey M: β = –1,741, *t*(32) = 6.863, *p* = 9.156 × 10^−8^; Monkey P: β = –389.6, *t*(20) = –5.435, *p* = 2.550 × 10^−5^), reflecting the increasing difference in PSE between forward and backward self-motion as self-motion amplitude increased. These data are consistent with the flow-parsing hypothesis, which predicts that the direction and magnitude of the bias induced by optic flow are determined by self-motion direction and amplitude, respectively.

To generate a single value that captures the perceptual bias for each self-motion amplitude, we further calculated a PSE shift as the difference in PSEs between forward and backward self-motion at each self-motion amplitude. We then scaled these PSE shifts by the sign of the object's horizontal location such that they would be positive if biases were in the direction predicted by flow parsing. We used linear regression to test the effect of self-motion amplitude on PSE shifts. Self-motion amplitude could account for approximately 86% of the variability in PSE shifts in Monkey M (*R*^2^ = 0.8597, *F*(1, 16) = 98.1, *p* = 3.14 × 10^−8^), as well as 65% in Monkey P (*R*^2^ = 0.6526, *F*(1, 10) = 18.8, *p* = 1.48 × 10^−3^). In both monkeys, self-motion amplitude was assigned a significant positive β weight (Monkey M: β = 1741, *t*(16) = 9.90, *p* = 3.14 × 10^−8^; Monkey P: β = 390, *t*(10) = 4.33, *p* = 1.48 × 10^−3^), indicating that faster self-motion induced larger biases in perceived object direction.

**Figure 5.**
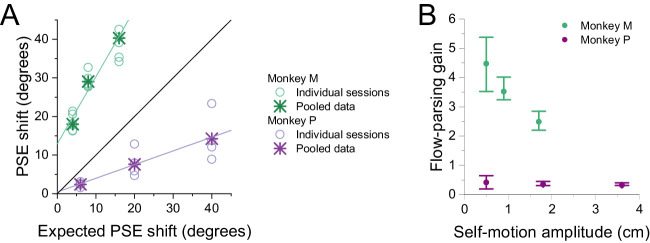
Summary of the relationship between measured and predicted PSE shifts. (A) Both monkeys show a strong, roughly linear relationship between measured PSE shifts and those predicted by flow parsing. Circles: PSE values from individual sessions for Monkey M (green) and Monkey P (purple). Asterisks: PSE shifts from psychometric data that were pooled across sessions. (B) Average flow-parsing gain (observed PSE shift divided by expected PSE shift), as a function of self-motion amplitude, for Monkey M (green) and Monkey P (purple). Error bars indicate 95% confidence intervals.

To assess whether the magnitude of PSE shifts is consistent with predictions of flow parsing, we compared measured PSE shifts with predicted shifts that are based on the assumption of complete flow parsing (see Methods for details). Figure 5A shows that measured PSE shifts are strongly correlated with predicted shifts (linear regression, Monkey M: β = 1.74, *t*(16) = 9.90, *p* = 3.14 × 10^−8^; Monkey P: β = 0.355, *t*(10) = 4.33, *p* = 1.49 × 10^−3^). Monkey M demonstrates greater PSE shifts than predicted by flow parsing, as indicated by a β value of 1.74, while Monkey P exhibits smaller than expected PSE shifts, as indicated by a β value of 0.355. Thus, while both animals show PSE shifts that are strongly correlated with predictions of flow parsing, they have very different ratios of observed to predicted values. Moreover, as discussed below, these ratios changed somewhat over a long time scale as animals performed different experiments.

For each self-motion amplitude, we computed a flow-parsing gain (see Methods), which is defined as the ratio of measured to predicted PSE shifts. If there is a strictly proportional relationship between measured and predicted PSE shifts, then flow-parsing gains should be constant across self-motion amplitudes. This is approximately the case for Monkey P but is clearly not true for Monkey M (Figure 5B). For Monkey M, flow-parsing gains decrease substantially as self-motion amplitude increases. Since PSE shifts should decrease toward zero as self-motion amplitude approaches zero, the flow-parsing gains for Monkey M suggest that the relationship between measured and predicted PSE shifts is likely to be nonlinear for self-motion amplitudes smaller than those that we measured.

Flow parsing requires signals related to self-motion to interact with signals related to object motion. Since adding self-motion signals into the computation of object direction should add noise, we expected that psychophysical thresholds would increase with self-motion amplitude. Indeed, we found this to be the case (Figure 4E and F). To quantify this effect, we combined data across self-motion directions (including stationary background trials) and performed a linear regression of threshold, *σ* as defined in Equation 2, onto self-motion amplitude. This regression revealed that self-motion amplitude is significantly predictive of thresholds (Monkey M: β = 877.0, *t*(40) = 6.594, *p* = 6.921 × 10^−8^; Monkey P: β = 77.96, *t*(26) = 4.057, *p* = 4.027 × 10^−4^), while there is no significant difference in thresholds between forward and backward self-motion conditions (Wilcoxon rank-sum test; Monkey M: *Z* = 0.4588, *p* = 0.646; Monkey P: *Z* = –0.953, *p* = 0.341). These data support the expectation that flow parsing adds noise to the representation of object direction.

## Experiment 2: Effect of object location

For our task, which involves discriminating the horizontal component of object motion, the flow-parsing hypothesis predicts that optic flow should only affect the subject's responses if there is a horizontal component of optic flow at the location of the object. Thus, the flow-parsing hypothesis predicts that biases in perceived object direction should depend on the horizontal location of the object but not its vertical location (Equation 1). When the object is located directly above the fixation point, optic flow at the location of the object is vertical and should not bias perceived object direction.

**Figure 6.**
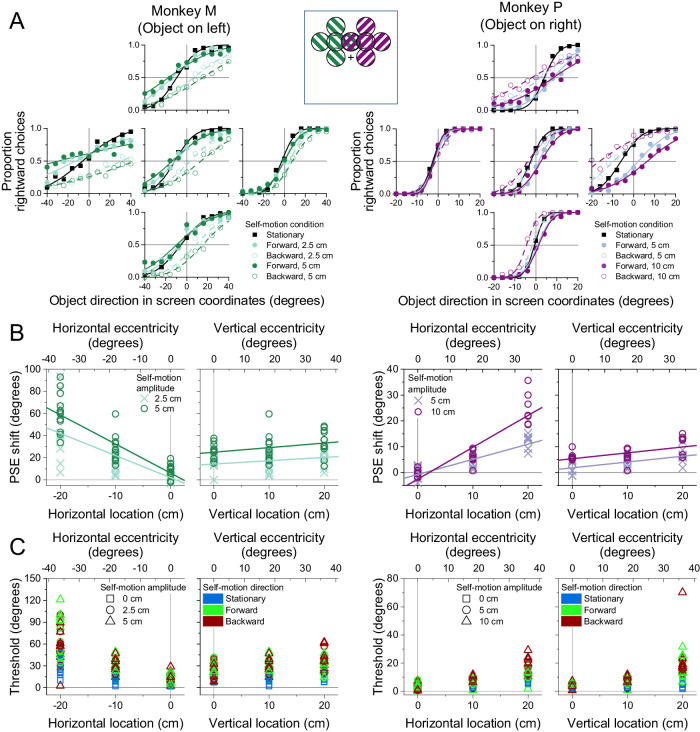
Dependence of discrimination performance on horizontal and vertical object location. (A) Psychometric functions (format as in Figure 4A and B) for each animal at five different object locations (17,382 trials from Monkey M; 16,996 trials from Monkey P). Inset: Schematic of object locations tested for Monkey M (green) and Monkey P (purple). (B) PSE shifts are plotted as a function of horizontal and vertical object location for each animal. Each datum represents a PSE shift from a single session. Circles and crosses denote two different amplitudes of self-motion that were tested. Lines show regression fits (see text for details). (C) Psychophysical thresholds are plotted as a function of horizontal and vertical object location. Symbol shape denotes self-motion amplitude, and symbol color denotes self-motion condition (blue: stationary; green: forward; red: backward).

In this experiment, the object was placed at one of five locations in the visual field (Figure 6A, inset). These include three locations with the same vertical coordinate and varying horizontal positions, as well as three locations with the same horizontal coordinate and varying vertical positions.

### Methods

The same two monkeys participated in this experiment as in Experiment 1. The object was placed in one of five positions on the screen (cross-shaped configuration), centered on (–10 cm, 10 cm) for Monkey M and (10, 10) for Monkey P (Figure 6A, inset). The object locations were spaced in increments of 10 cm (which corresponds to 17.5° for Monkey M and 16.9° for Monkey P, due to small differences in viewing distance for the two animals). The object's diameter was 8 cm, and its amplitude of motion over the trial was 15 cm.

Optic flow simulated forward or backward self-motion at one of two velocities. Self-motion amplitudes were 2.5, 5 cm for Monkey M and 5, 10 cm for Monkey P. A condition with no self-motion (stationary background dots) was interleaved with these self-motion conditions. Across trials, the mask was always centered on the object's position, and its diameter was 1.5 times the object's diameter. Both the mask and the aperture for object motion remained stationary in screen coordinates throughout each trial.

We tested 5 object locations, 11 object directions, and 5 self-motion conditions (two amplitudes each of forward and backward self-motion, plus one stationary condition). All 275 distinct conditions were interleaved within a single block of trials in each session. Monkey M completed 17,382 trials over 12 sessions, and Monkey P completed 16,996 trials over 8 sessions.

### Results

Figure 6A displays the subjects’ psychometric functions for the five different object locations. From these psychometric functions, it is apparent that perceptual biases increase substantially with the object's horizontal distance away from the vertical meridian. When the object was in the left visual field (Figure 6A, left, Monkey M), biases increased as the object was positioned further to the left, as demonstrated by the increasing separation between psychometric curves for the forward and backward self-motion conditions. When the object was in the right visual field (Figure 6A, right, Monkey P), biases increased as the object was positioned further to the right. There is little, if any, visible effect of the object's vertical position on perceptual biases, although psychophysical thresholds tended to increase with distance away from the horizontal meridian, as quantified below.

The effect of an object's horizontal or vertical position on directional biases induced by flow parsing is summarized in Figure 6B. Multiple regressions of PSE shift onto horizontal distance from the vertical meridian, vertical location, and self-motion amplitude demonstrated strong relationships in both subjects (Monkey M: *R*^2^ = 0.621, *F*(5, 114) = 37.4, *p* = 1.53 × 10^−22^; Monkey P: *R*^2^ = 0.794, *F*(5, 74) = 57.0, *p* = 5.24 × 10^−24^). There was a significant main effect of horizontal location on PSE shifts (Monkey M: β = 231.5, *t*(114) = 12.603, *p* = 3.263 × 10^−23^; Monkey P: β = 90.914, *t*(74) = 14.61, *p* = 3.191 × 10^−23^). In one subject, there was also a strong interaction between horizontal location and self-motion amplitude (Monkey P: β = 1,295, *t*(74) = 5.202, *p* = 1.780 × 10^−6^), whereas this interaction only approached significance in the other subject (Monkey M: β = 2,508, *t*(114) = 1.707, *p* = 0.09055). The effect of the object's horizontal position on biases in perceived object direction is consistent with predictions of the flow-parsing hypothesis.

The flow-parsing hypothesis predicts no effect of the object's vertical position on perceptual biases (Equation 1). However, we observed a main effect of vertical location on PSE shifts that was significant in one animal (Monkey P: β = 22.534, *t*(74) = 3.620, *p* = 5.438 × 10^−4^) and borderline in the other (Monkey M: β = 35.51, *t*(114) = 1.934, *p* = 0.0557). The effect of the object's vertical position on horizontal perceptual biases suggests an imperfect compensation for self-motion that may be augmented by the overall magnitude of optic flow, even if components of the optic flow are in a direction irrelevant to the discrimination. Importantly, the coefficients associated with vertical position were several fold smaller than those associated with horizontal position, and this much greater effect of horizontal location is roughly in line with the predictions of the flow-parsing hypothesis. Further, there was no significant interaction between vertical location and self-motion amplitude (Monkey M: *t*(114) = 0.3099, *p* = 0.7573; Monkey P: *t*(74) = 0.01668, *p* = 0.9867), suggesting that the overall vertical magnitude of optic flow does not have the same effect on object direction perception as does the horizontal magnitude.

The object's location may also be expected to affect the subjects’ discrimination thresholds, as sensitivity to direction and speed decreases as stimuli become more peripheral (Orban, Van Calenbergh, De Bruyn, & Maes, 1985; Van de Grind, Van Doorn, & Koenderink, 1983). Indeed, we find that psychophysical thresholds increase with the horizontal and vertical eccentricity of the object's location (Figure 6C). We performed a multiple regression of thresholds onto horizontal distance from the vertical meridian, vertical position, and self-motion amplitude, demonstrating strong relationships in both subjects (Monkey M: *R*^2^ = 0.537, *F*(5, 294) = 68.1, *p* = 4.08 × 10^−47^; Monkey P: *R*^2^ = 0.553, *F*(5, 194) = 47.93, *p* = 3.928 × 10^−32^). There was a significant main effect of horizontal location on thresholds (Monkey M: β = 224.82, *t*(294) = 16.07, *p* = 4.538 × 10^−42^; Monkey P: β = 46.22, *t*(194) = 8.337, *p* = 1.448 × 10^−14^), as well as a significant main effect of self-motion amplitude (Monkey M: β = 345.8, *t*(294) = 7.311, *p* = 2.552 × 10^−12^; Monkey P: β = 60.78, *t*(194) = 6.487, *p* = 7.271 × 10^−10^). We also observed a significant main effect of vertical location on thresholds (Monkey M: β = 34.83, *t*(294) = 2.490, *p* = 0.01334; Monkey P: β = 52.94, *t*(194) = 9.549, *p* = 6.310 × 10^−18^). There was a significant interaction between horizontal location and amplitude (Monkey M: β = 3,295.4, *t*(294) = 4.4066, *p* = 1.473 × 10^−5^; Monkey P: β = 437.0, *t*(194) = 2.950, *p* = 3.576 × 10^−3^), and the interaction between vertical location and amplitude was either significant or approaching significance (Monkey M: β = 1,356, *t*(294) = 1.814, *p* = 0.07077; Monkey P: β = 787.24, *t*(194) = 5.313, *p* = 2.966 × 10^−7^). Overall, the thresholds clearly suggest that object direction discrimination is less sensitive when there is a longer optic flow vector to parse at the location of the object, no matter whether that vector length is manipulated by object location or by self-motion amplitude.

## Experiment 3: Effect of object distance

Experiment 2 demonstrated that the size of the flow-parsing effect depends on the object's location relative to the flow field, within the frontoparallel plane. The question remains as to whether the visual system uses information about three-dimensional scene structure to compute an object's motion during self-motion. If it does, then we would expect the biases induced by optic flow to depend on the distance of the object from the observer.

Equation 1 indicates that the retinal magnitude of an optic flow vector is inversely related to its distance from the observer. This is because the horizontal and vertical fields of view encompass more space at distant depths. Therefore, far objects appear smaller on the retina, and a far object will move more slowly on the retina relative to a near object moving at the same world-relative speed. If two objects at different depths at the same retinal location move with the same world-relative velocity, the near object will produce greater retinal image motion. However, the magnitude of optic flow vectors at the same depths as these objects will scale with distance in the same way as object velocity on the retina. Therefore, subtraction of optic flow vectors at the respective depths of a near and a far object would be expected to have the same effect on their perceived direction, if the near and far objects have the same velocity relative to the world.

**Figure 7.**
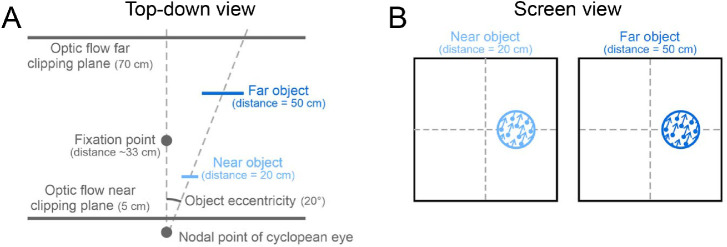
Schematic of stimulus manipulations for simulating different object distances. (A) A top-down view of the stimulus. The far object was larger and farther away from the midline in world coordinates, relative to the near object. These changes kept the object's size and position constant in retinal coordinates. (B) Image view of the stimulus (background dots are not shown for clarity). The object's size, velocity, and position were kept constant in retinal coordinates such that binocular disparity was the only cue to depth.

In contrast, if two objects have the same retinal velocity, the farther object must be moving faster relative to the world. As a result, subtraction of the optic flow vector at the object's three-dimensional location should produce a smaller perceptual bias for a far object than for a near object. In this experiment, we manipulated the apparent distance of the object by manipulating binocular disparity cues while keeping the retinal location, size, and speed constant (Figure 7). Correspondingly, the object's location, size, and speed in the world covaried with the depth specified by binocular disparity. If the visual system uses disparity information about the three-dimensional position of the object in the computation of flow parsing, we would expect larger biases in perceived direction for the near object than for the far object.

### Methods

The same two monkeys participated in this experiment as in Experiments 1 and 2. The monkeys discriminated the direction of motion of an object that was placed at one of two depths as defined by binocular disparity (Figure 7). The object was positioned on the horizontal meridian 20° to the left of fixation for Monkey M and 20° to the right of fixation for Monkey P. The object was simulated to lie at a distance of either 20 cm or 50 cm. To maintain a constant retinal eccentricity, the far object's location was more eccentric relative to the world (Figure 7).

To ensure that binocular disparity was the only cue to the object's depth, pictorial depth cues were abolished. The object's diameter, along with star size and density, were scaled appropriately as a function of distance, in order to keep retinal size and density constant. The object's diameter was scaled such that it would be 10 cm if it were at the depth of the subjects’ fixation (subtending 17.5° for Monkey M and 16.9° for Monkey P). The object's spread in depth remained constant at 0.1 cm. The object's world-relative speed was also scaled with distance so that the object would produce the same retinal image motion regardless of its depth. Its retinal speed corresponded to world-relative object amplitudes of 7.5 cm for Monkey M and 15 cm for Monkey P if it were at the depth of fixation.

In this experiment, the cloud of background dots extended between 5 cm and 70 cm in depth from the subject. This extension ensured that far objects would be embedded in background dots rather than near the back of the dot cloud. As with the previous experiment, self-motion amplitudes were 2.5, 5 cm for Monkey M, and 5, 10 cm for Monkey P. A condition with stationary background dots was incorporated for both object depths.

Altogether, this experiment included 2 object depths, 11 unique object directions, and 5 self-motion conditions (two speeds each of forward and backward self-motion, plus one stationary condition), for a total of 110 unique stimulus conditions. Monkey M completed four sessions in which the two object depths were interleaved within a block; otherwise, trials were split into blocks by object depth. Subjects completed one block of each object depth within each session, and the order of the blocks was counterbalanced between sessions. Monkey M completed 9,350 trials over eight sessions, and Monkey P completed 11,880 trials over eight sessions.

### Results

**Figure 8.**
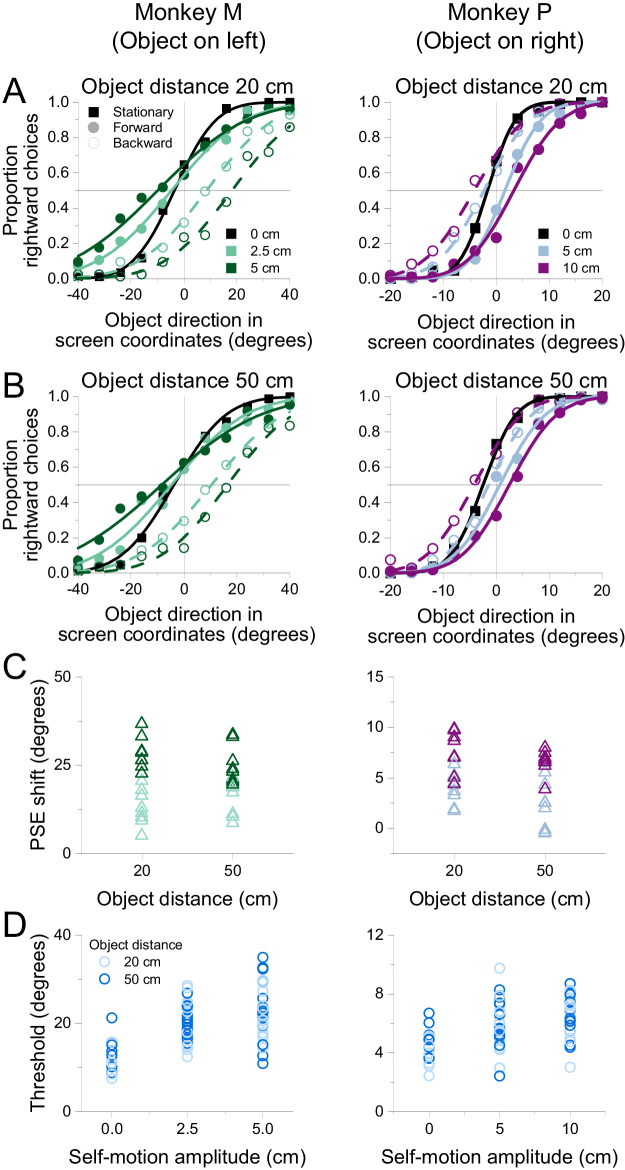
Dependence of direction discrimination performance on simulated distance. (A) Summary psychometric curves for Monkeys M (left, 9,350 trials) and P (right, 11,880 trials) for an object distance of 20 cm. Format as in Figure 4A and B. (B) Summary psychometric functions for the object distance of 50 cm. (C) PSE shifts are plotted as a function of object distance for each animal. Colors denote self-motion amplitude variations, as in Panels A and B. (D) Psychophysical thresholds are plotted as a function of self-motion amplitude. Lighter and darker colors denote near and far object distances, respectively.

Figure 8 illustrates summary psychometric functions that describe the performance of the two subjects at discriminating the direction of a near object (Figure 8A) and a far object (Figure 8B) in the presence of optic flow. The psychometric curves are similar between the two simulated distances, suggesting that object depth, as defined by binocular disparity, is not a major factor in determining the extent of flow parsing.

PSE shifts are displayed as a function of object distance in Figure 8C. We performed a multiple linear regression of PSE shift onto the object's distance and self-motion amplitude, which accounted for a substantial fraction of variance in both subjects (Monkey M: *R*^2^ = 0.6437, *F*(3, 28) = 16.86, *p* = 1.879 × 10^−6^; Monkey P: *R*^2^ = 0.6256, *F*(3, 28) = 15.59, *p* = 3.722 × 10^−6^). There was a main effect of self-motion amplitude on PSE shifts for both animals (Monkey M: β = 493.4, *t*(28) = 6.894, *p* = 1.711 × 10^−7^; Monkey P: β = 82.98, *t*(28) = 6.424, *p* = 5.891 × 10^−7^), indicating larger biases during faster self-motion. The main effect of object distance on PSE shifts was modest, with a weakly significant dependence in only one subject (Monkey P: β = 4.851, *t*(28) = 2.253, *p* = 0.03225; Monkey M: *t*(28) = 0.1150, *p* = 0.9093), and no significant interactive effect of object distance and self-motion amplitude for either animal (Monkey M: *t*(28) = 1.746, *p* = 0.09163; Monkey P: *t*(28) = –0.6561, *p* = 0.5171). While the effects of distance are weak and inconsistent, there is a modest tendency for perceptual biases to decrease with distance, especially for Monkey P. The weakness of this effect suggests that the visual system does not use all of the available three-dimensional information from the scene to perform flow parsing and compute object motion relative to the world. The visual system might perform an approximate form of flow parsing by subtracting optic flow vectors at the same retinal position as the object but over a range of depths that is not well matched to the object's distance. Alternatively, flow parsing might rely on other depth-related cues (e.g., size, speed) that were placed in conflict with binocular disparity in this experiment, thus limiting the effect observed.

We also found no clear effect of object distance on direction discrimination thresholds (Figure 8D). A multiple linear regression of thresholds against self-motion amplitude and object distance indicated a significant relationship in both subjects (Monkey M: *R*^2^ = 0.357, *F*(3, 76) = 14.05, *p* = 2.252 × 10^−7^; Monkey P: *R*^2^ = 0.2571, *F*(3, 76) = 8.768, *p* = 4.589 × 10^−5^). However, only self-motion amplitude was significantly predictive of thresholds (Monkey M: β = 192.9, *t*(76) = 6.371, *p* = 1.309 × 10^−8^; Monkey P: β = 21.77, *t*(76) = 4.979, *p* = 3.885 × 10^−6^). There was no significant main effect of object distance (Monkey M: *t*(76) = –1.235, *p* = 0.2207; Monkey P: *t*(76) = –0.9069, *p* = 0.3673) or a significant interaction between object distance and self-motion amplitude (Monkey M: *t*(76) = –0.1468, *p* = 0.8837; Monkey P: *t*(76) = 0.8298, *p* = 0.4092). This result is compatible with the very limited effect of object distance on PSE shifts.

## Experiment 4: Effect of vestibular self-motion cues

In human psychophysical experiments, flow-parsing gains were found to vary across subjects but were consistently less than unity (Dokka et al., 2015; Fajen et al., 2013; Layton & Niehorster, 2019; Niehorster & Li, 2017), which was also the case for one of our two animals. While it is possible that people do not completely compensate for self-motion when judging object motion under natural conditions, it is also possible that optic flow alone, in the absence of nonvisual (e.g., vestibular) signals that normally accompany real self-motion, leads to an underestimation of self-motion velocity due to cue conflict. If so, then addition of nonvisual self-motion signals might result in stronger flow-parsing effects.

In this experiment, we added physical translation of the subjects to optic flow, thus providing congruent and coherent vestibular and visual self-motion cues. Because it is known that both human and monkey subjects integrate visual and vestibular self-motion cues to judge heading (Butler et al., 2010; Fetsch et al., 2009; Gu et al., 2008), we hypothesized that adding platform motion to optic flow would allow subjects to better compensate for self-motion when judging object motion. We expected this to manifest as larger PSE shifts and therefore greater flow-parsing gains.

### Methods

The same two subjects participated in this experiment as in Experiments 1 to 3.

The object was positioned on the horizontal meridian, 20° to the left of fixation for Monkey M and 20° to the right of fixation for Monkey P. The object's diameter was 10 cm, and its amplitude of motion was 5 cm for Monkey M and 10 cm for Monkey P. The object's amplitude differed between subjects because the subjects had been trained on different self-motion amplitudes, and we wished to keep the relationship between self-motion velocity and object velocity—and therefore the expected PSE shift—consistent across animals.

This experiment introduced variation in the nature of the self-motion cues being presented to the subjects. In the *visual* condition, optic flow was presented as the only cue to self-motion, as in the other experiments described above. In *visual + vestibular* trials, optic flow was accompanied by platform motion. Because the screen was mounted on the platform, it remained at a constant distance from the monkey during each trial; thus, object motion in screen (or retinal) coordinates was the same for visual and visual + vestibular conditions. Platform motion matched the velocity profile of translation simulated by optic flow, creating a coherent multisensory percept of self-motion. As with Experiments 2 and 3, self-motion amplitudes were 2.5, 5 cm for Monkey M and 5, 10 cm for Monkey P.

This experiment interleaved 2 stimulus modalities (visual, visual + vestibular), 5 self-motion velocities (two forward, two backward, one stationary), and 11 object directions. All 110 stimulus conditions were interleaved within a single block of trials per session. Because the stimulus was identical between visual and visual + vestibular conditions for the stationary self-motion condition, stationary trials were pooled in analyses. Monkey M completed 15,730 trials over 12 sessions, and Monkey P completed 14,300 trials over 8 sessions.

### Results

**Figure 9.**
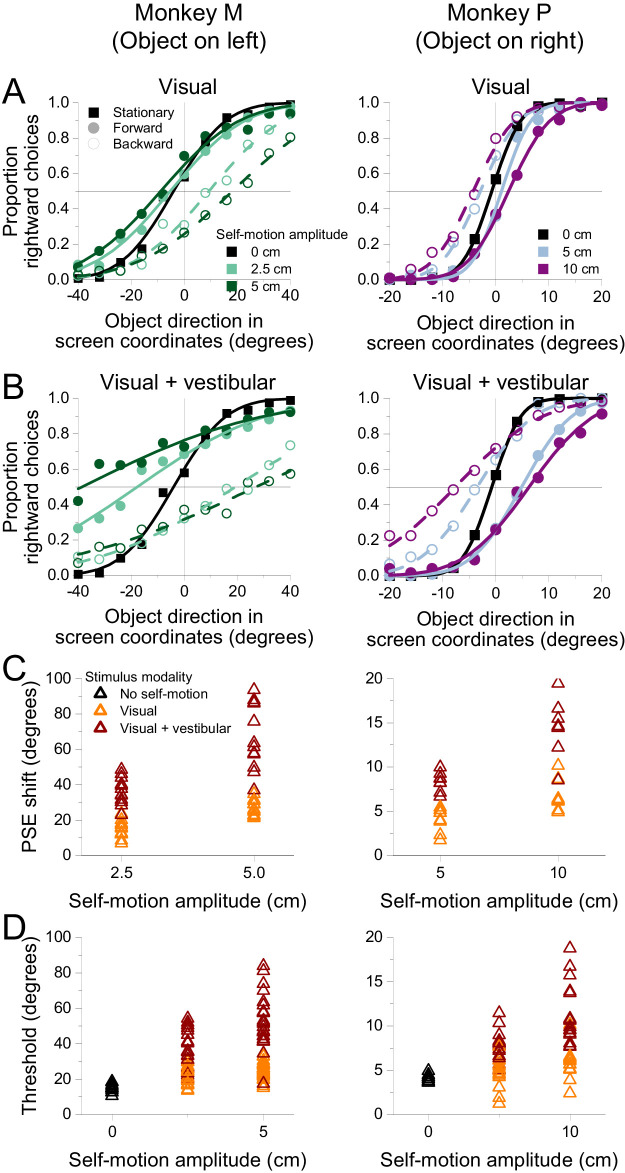
Adding vestibular self-motion cues to optic flow increases biases in perceived object direction. (A) Summary psychometric curves for Monkeys M (left, 15,730 trials) and P (right, 14,300 trials) when self-motion is indicated solely by optic flow. Format as in Figure 4A and B. (B) Summary psychometric curves for interleaved conditions in which self-motion is indicated by both optic flow and vestibular signals. Format as in Panel A. (C) PSE shifts are plotted as a function of self-motion amplitude. Each datum represents a PSE shift from a single session, and colors denote the modality of self-motion cues (orange: visual; red: visual + vestibular). (D) Psychophysical thresholds are plotted as a function of self-motion amplitude. Colors denote the self-motion condition (black: stationary; orange: visual; red: visual + vestibular).

Figure 9 shows psychometric functions of the two subjects for the visual (Figure 9A) and visual + vestibular (Figure 9B) conditions. In both subjects, the biases induced by self-motion were greater when vestibular self-motion cues were added to optic flow, as summarized in Figure 9C. We performed multiple linear regressions of PSE shift onto stimulus modality and self-motion amplitude and found good predictive power for both subjects (Monkey M: *R*^2^ = 0.7870, *F*(3, 44) = 54.19, *p* = 8.050 × 10^−15^; Monkey P: *R*^2^ = 0.8045, *F*(3, 28) = 38.41, *p* = 4.662 × 10^−10^). We found a highly significant main effect of stimulus modality on PSE shifts (Monkey M: β = 31.07, *t*(44) = 10.22, *p* = 3.412 × 10^−13^; Monkey P: β = 6.044, *t*(44) = 8.457, *p* = 3.394 × 10^−9^), as well as moderate evidence for an interaction between stimulus modality and self-motion amplitude (Monkey M: β = 733.5, *t*(44) = 3.016, *p* = 4.248 × 10^−3^; Monkey P: β = 67.78, *t*(28) = 2.371, *p* = 0.02487). Additionally, there was a main effect of self-motion amplitude on PSE shifts (Monkey M: β = 485.0, *t*(44) = 2.820, *p* = 7.174 × 10^−3^; Monkey P: β = 54.35, *t*(28) = 2.688, *p* = 0.01195).

**Figure 10.**
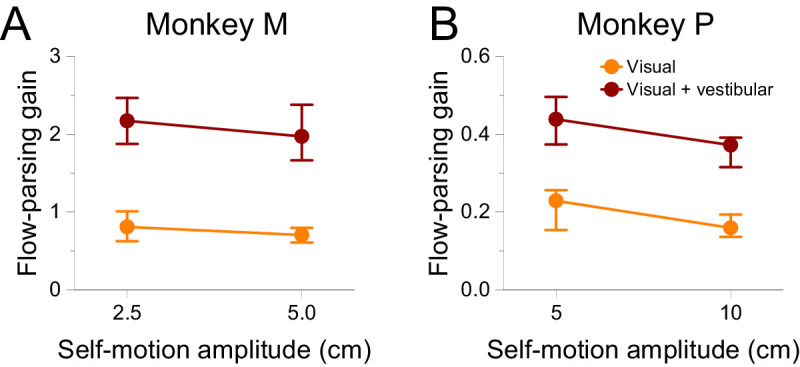
Adding vestibular self-motion cues to optic flow increases flow-parsing gains. (A) Summary of flow-parsing gains for Monkey M. Symbol color denotes the modality of self-motion cues (orange: visual; red: visual + vestibular). Error bars indicate 95% confidence intervals. (B) Summary of flow-parsing gains for Monkey P. Format as in Panel A.

The data of Figure 9C appear to be consistent with vestibular stimulation scaling the subjects’ flow-parsing gains across self-motion amplitudes, as indicated by replotting these data as flow-parsing gains in Figure 10. Multiple linear regressions of flow-parsing gains onto stimulus modality and self-motion amplitude revealed strong relationships in both subjects (Monkey M: *R*^2^ = 0.7405, *F*(3, 44) = 41.86, *p* = 6.012 × 10^−13^; Monkey P: *R*^2^ = 0.7644, *F*(3, 28) = 30.28, *p* = 6.216 × 10^−9^). The presence of vestibular self-motion cues was strongly predictive of flow-parsing gains (Monkey M: β = 1.086, *t*(44) = 11.116, *p* = 2.316 × 10^−14^; Monkey P: β = 0.2117, *t*(28) = 9.257, *p* = 5.140 × 10^−10^), indicating greater flow-parsing gains when vestibular self-motion cues were presented. There was no significant main effect of self-motion amplitude on flow-parsing gains (Monkey M: *t*(44) = –0.5348, *p* = 0.5955; Monkey P: *t*(28) = –1.116, *p* = 0.2739), nor was there a significant interaction between stimulus modality and self-motion amplitude (Monkey M: *t*(44) = –0.5516, *p* = 0.5840; Monkey P: *t*(28) = –0.6070, *p* = 0.5487). These results suggest that vestibular self-motion cues increase flow-parsing gains but that flow-parsing gains stay fairly consistent across self-motion amplitudes.

The subjects’ discrimination thresholds are presented as a function of self-motion amplitude in Figure 9D. Multiple linear regressions indicated that stimulus modality and self-motion amplitude accounted for substantial variance in thresholds (Monkey M: *R*^2^ = 0.6822, *F*(3, 104) = 74.41, *p* = 8.812 × 10^−26^; Monkey P: *R*^2^ = 0.6234, *F*(3, 68) = 37.52, *p* = 2.012 × 10^−14^). The main effect of self-motion amplitude was either significant or approached significance (Monkey M: β = 191.2, *t*(104) = 3.059, *p* = 2.822 × 10^−3^; Monkey P: β = 17.09, *t*(68) = 1.954, *p* = 0.05480), consistent with results of Experiment 1. There was also a significant main effect of stimulus modality on thresholds (Monkey M: β = 20.52, *t*(104) = 11.28, *p* = 9.301 × 10^−20^; Monkey P: β = 3.837, *t*(68) = 7.535, *p* = 1.533 × 10^−10^), reflecting higher thresholds overall when platform motion was combined with optic flow. A significant interaction between stimulus modality and self-motion amplitude (Monkey M: β = 402.8, *t*(104) = 3.306, *p* = 1.299 × 10^−3^; Monkey P: β = 53.51, *t*(68) = 3.138, *p* = 2.513 × 10^−3^) indicates that increasing self-motion amplitude causes a greater increase in threshold when vestibular self-motion cues are present than when only optic flow is present. Because PSE shifts depend on self-motion amplitude and the interaction between amplitude and stimulus modality in a similar manner, it is possible that the amount of noise present in the flow-parsing process depends on the magnitude of the subtraction taking place.

## Changes in flow-parsing gains over time

**Figure 11.**
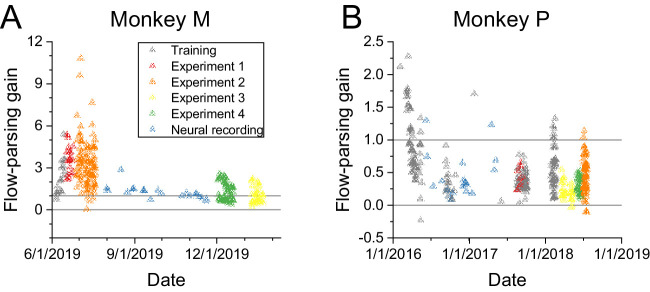
Flow-parsing gains decreased gradually over time. (A) Monkey M's flow-parsing gains over time. Horizontal reference lines at 0 and 1 indicate gains corresponding to no flow parsing (retinal coordinates) and complete flow parsing (world-centered coordinates), respectively. Colors indicate the experiment from which the flow-parsing gains resulted (red: Experiment 1, orange: Experiment 2, yellow: Experiment 3, green: Experiment 4). Additional flow-parsing gains were collected from training sessions (gray) and neural recordings (blue), to be described elsewhere. (B) Monkey P's flow-parsing gains over time. Colors and reference lines as in Panel A.

Comparison of Figure 10 (visual condition) with Figure 5B clearly reveals that flow-parsing gains were generally lower during Experiment 4 than during Experiment 1, especially for Monkey M. These differences turn out to be mainly effects of time rather than the specific stimulus manipulations used. Both monkeys demonstrated a decrease in flow-parsing gains over time, compensating less for their self-motion in later sessions (Figure 11). Monkey M's flow-parsing gains were well above 1 in Experiments 1 and 2, indicating overcompensation for self-motion when judging object motion, but they decreased toward 1 in later experiments (Figure 11A). Monkey P's flow-parsing gains started closer to 1 and decreased with time (Figure 11B), resulting in an undercompensation for self-motion. Note, however, that the time scale is much longer for Monkey P (Figure 11B) than for Monkey M (Figure 11A). Monkey P underwent months of training in the task before neural recordings (to be described elsewhere) were performed, and the behavioral experiments described here were subsequently performed much later. Thus, for Monkey P, the behavioral tests described here were done after flow-parsing gains had largely stabilized. In contrast, for Monkey M, behavioral studies described here commenced shortly after the animal was well trained to perform the task with stationary background dots. Thus, during the time period over which behavioral data were collected for this study, with neural recordings done between Experiments 1–2 and Experiments 3–4, flow-parsing gains declined considerably. This makes it difficult to compare the magnitude of flow-parsing gains between experiments and monkeys; however, all of our main conclusions rely on comparisons made within an experiment for each animal.

**Figure 12.**
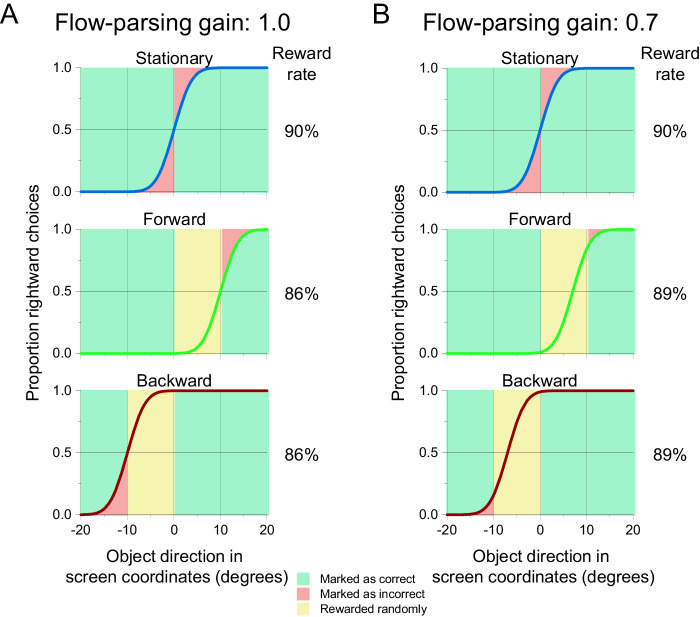
Reward paradigm may lead to decrease in flow-parsing gains over time. (A, B) Idealized psychometric functions corresponding to flow-parsing gains of 1.0 (A) and 0.7 (B). Shading indicates proportions of trials that are scored veridically as correct (green) or incorrect (red), as well as trials that are rewarded randomly (yellow; see Methods for details of reward regime). Psychometric curves for stationary (blue), forward (green), and backward (red) self-motion conditions are separated for clarity. The expected PSEs (assuming a flow-parsing gain of 1.0) for forward and backward self-motion are 10° and –10°, respectively. (A) Top row: When there is no self-motion, accuracy is determined by the proportion of trials in which the observer correctly indicates the object's motion in retinal (or screen) coordinates (green). Middle row: During forward self-motion, object directions between 0° and 10° are rewarded randomly, regardless of the subject's response (yellow). Bottom row: During backward self-motion, object directions between 0° and –10° are rewarded randomly (yellow). (B) When the flow-parsing gain is reduced to 0.7, the smaller PSE shifts lead to smaller areas in the forward and backward self-motion conditions that are marked as incorrect (red), resulting in an overall higher reward rate.

A possible explanation for the gradual reduction in flow-parsing gains over time could be the variable reward scheme that we used. Because we did not wish to encourage the monkeys to report the object's motion in one coordinate frame over the other, we randomly rewarded trials when the correct answer was inconsistent between the two coordinate frames. However, this reward strategy yields slightly higher overall reward rates when flow-parsing gains are smaller (Figure 12). Thus, the monkeys may have gradually learned that they were more likely to receive a reward for reporting the object's motion in retinal coordinates, and they may have adjusted their strategy accordingly over time.

Finally, it is also worth noting that the monkeys exhibited biases in perceiving object direction from the very first day of viewing stimuli in which the background contained optic flow that simulated forward or backward self-motion. Despite never being rewarded for such biases, the animals exhibited flow parsing spontaneously, with the largest effects occurring during the earliest sessions. This suggests that flow parsing is an automatic process that does not require any training.

## General discussion

The flow-parsing hypothesis accurately predicts several features of object motion perception during self-motion. Optic flow induces a bias in object motion perception (in retinal coordinates) that is consistently in the opposite direction to the optic flow directly surrounding the object. This effect remains under conditions that have opposite directions of optic flow surrounding the object, such as forward and backward self-motion or objects in the left or right visual hemifield. Furthermore, the size of the bias depends on the magnitude of the optic flow vector to subtract at the location of the object. Biases increase as self-motion speed increases and as the object's retinal eccentricity increases. Because our task involves discriminating the horizontal component of object direction, biases depend primarily on the horizontal eccentricity of the object, as predicted; however, we did also observe a relatively weak dependency of biases on vertical eccentricity. Counter to the prediction of the flow-parsing hypothesis, we found only a weak dependence, in one subject, of perceptual biases on an object's depth when specified by binocular disparity. When optic flow is supplemented with vestibular self-motion signals, flow-parsing biases increase, suggesting a facilitatory effect of multisensory signals on flow parsing. Across experiments, discrimination thresholds generally increased under the same conditions that increased biases, suggesting that the noise in the flow-parsing computation may depend on the magnitude of the optic flow vector to subtract.

While the flow-parsing gains of both animals declined over time before largely stabilizing, the gains of Monkey M were consistently about threefold greater than the gains of Monkey P (Figure 11). The reasons for this large difference between animals remain largely unclear. For Monkey P, flow-parsing gains started close to unity and may have declined as the animal gradually learned that the perceptual biases reduced reward rate and attempted to compensate for this effect. For Monkey M, the major unresolved question is why flow-parsing gains started out much greater than unity before declining over time. One possibility is that Monkey M substantially overestimated his self-motion velocity from optic flow, thus causing the neural mechanisms to compensate for larger flow vectors than would have actually been present at the location of the object.

Overall, our findings indicate that rhesus monkeys generally exhibit similar effects of optic flow on object motion perception as humans. Our experiments incorporated novel manipulations not previously examined in humans, such as the inclusion of vestibular self-motion signals, the effect of different self-motion speeds, and variation of both horizontal and vertical object positions, demonstrating that biases were sensitive to factors that affected the magnitude of the optic flow to be subtracted from an object's retinal motion. In addition to extending our understanding of perceptual hallmarks of flow parsing, our findings have built a framework for using monkeys as an animal model to investigate the neural correlates of flow parsing.

### Flow parsing of objects at varying depths

Our findings only partially agree with the flow-parsing hypothesis when we manipulated the object's depth as specified by binocular disparity, as only one subject had significantly greater biases when the object was nearer, and this effect was modest. This finding is inconsistent with previous experiments that found an effect of object depth on perceived object motion (Rushton et al., 2007; Rushton & Warren, 2005; P. A. Warren & Rushton, 2007, 2009b). A key difference between these previous experiments and ours is that the direction of simulated self-motion was lateral, and the object's depth was indicated by motion parallax in addition to binocular disparity. While disparity has been found to facilitate the detection of an independently moving object (Rushton et al., 2007), additional depth cues may be necessary to enable the accurate compensation for one's self-motion. In order to keep our object at a constant position and size on the retina, the object moved in depth along with the subject (like an insect crawling along your windshield while you are driving). This is clearly unnatural, as an object moving in a world-centered frontoparallel plane would get closer to the observer when they move forward. Correspondingly, the object's retinal position would become more eccentric, and its retinal velocity and size would increase. By confining an object to stay at a constant distance from the subject, potentially important depth cues are unavailable, and these depth cues might be used by the brain to discount self-motion when computing object velocity in the world.

Alternatively, it is possible that the visual system operates differently when parsing radial optic flow. During lateral self-motion and fixation on a world-fixed target (as in Rushton & Warren, 2005), the depth of an optic flow vector dictates its retinal direction, as points nearer than fixation move counter to the head while points farther than fixation move with the head. Flow parsing when judging a near object's motion should then induce biases in the opposite direction to those when judging a far object's motion. Conversely, during forward and backward self-motion, depth does not affect the vector's direction. The visual system might therefore approximate flow parsing by subtracting a two-dimensional optic flow field regardless of the object's depth. This strategy would result in an undercompensation for self-motion for objects nearer than the subtracted flow field and an overcompensation for objects farther than the subtracted flow field. Additional studies will be required to clarify how disparity and other depth cues modulate flow parsing across a broader range of conditions.

### Multisensory facilitation of flow parsing

Adding vestibular self-motion cues to optic flow enhances flow-parsing biases in a multiplicative manner, which is well described as an increase in flow-parsing gain. Unlike the other variables we manipulated in our experiments (self-motion amplitude, object location, object depth), the flow-parsing hypothesis does not predict a change in PSE shifts with the manipulation of stimulus modality, since flow parsing has been considered thus far a purely visual mechanism. However, our data show that the addition of vestibular signals affects the perceptual response to the same visual stimulus. One possible explanation for these increases in biases is that the integration of visual and vestibular signals produces a more accurate estimate of self-motion, which then modulates local neural responses to object motion. If so, then compensation for self-motion would be expected to generate a more accurate percept of object motion relative to the world, yielding flow-parsing gains closer to 1. While this was true for Monkey P, the addition of vestibular signals elevated the flow-parsing gains of Monkey M to well above 1, suggesting that this simple hypothesis is not correct. Rather, it appears that the addition of vestibular cues increases the estimates of self-motion velocity used to perform flow parsing, even if those estimates are already inflated.

It seems disadvantageous for vestibular self-motion signals to increase flow-parsing gains beyond 1, causing an observer to overcompensate for self-motion when judging object motion. Monkey M appeared to naturally overcompensate for his self-motion from the beginning of testing, showing flow-parsing gains much greater than 1 in Experiments 1 and 2 (Figure 11A). His overcompensation decreased over time, possibly reflecting a recalibration of estimated self-motion velocity based on optic flow. When platform motion was incorporated, he apparently could not ignore the effect of the new self-motion cue because he had not yet learned to do so. As a result, his flow-parsing gains increased toward his original gains in the visual condition, which were well above 1. Therefore, vestibular cues might facilitate flow parsing toward the subject's baseline amount of compensation. It may be useful to test this hypothesis on subjects that have not been trained out of their original flow-parsing gains.

### Limitations of the experiments

Some aspects of our stimuli were unnatural and may have affected the perceptual effects that we observed. One of these aspects was the use of dot motion within an aperture to represent object motion. Although one reason for this decision was to keep the object within receptive fields of neurons to be recorded during this flow-parsing task (in experiments to be described elsewhere), using apertured motion was also beneficial for psychophysical study. Because the border of the object stayed stationary on the screen, the monkey could only use dot motion rather than positional changes to discriminate motion direction successfully. Additionally, our experiments were designed without the assumption that complete flow parsing would occur naturally in monkeys. As a result, we manipulated object direction in screen/retinal coordinates and measured flow-induced effects as biases relative to a retinal reference frame. Had the object's border moved, the expected effect of flow parsing on perceived object direction would differ depending on the object's direction, due to the nonuniformity of radial optic flow fields. By removing the confounds introduced by a physically moving object, we could more directly test the effect of optic flow on perceived object motion. Also, in one animal, we confirmed that similar effects were seen when using an object whose boundaries moved naturalistically.

The world-relative movement of the object in depth along with the subject (Figure 2C) also simulated an uncommon situation in the natural world. Again, this design served purposes for both physiological and psychophysical experimentation. Had the object remained at a constant world-relative depth throughout the trial, its retinal position, disparity, size, and speed would have changed as the monkey moved forward or backward. Because we wished to test the effect of different background optic flow patterns on identical retinal object motion, we chose to keep these elements consistent by maintaining a constant distance between the object and the subjects within each trial. Thus, by sacrificing some naturalness, we gained a much cleaner test of the flow-parsing hypothesis.

A third unnatural facet of the stimulus was the circular mask that surrounded the object. Our primary motivation for this was to reduce the stimulation of inhibitory surrounds during neural recordings. However, the persistence of flow-parsing effects despite the removal of local motion contrast cues also supports the notion that flow parsing is largely a global subtraction process (P. A. Warren & Rushton, 2009a). In fact, all three unnatural facets of the stimulus that are discussed here would likely be expected to decrease the effect of optic flow on perceived object motion. The difference in the magnitude of effects between subjects may have arisen because each subject responded differently to the unnatural elements of the stimulus. Nevertheless, the persistence of perceptual biases (relative to a retinal reference frame) suggests that flow parsing is a robust phenomenon, and it may become even more effective as the stimulus becomes more naturalistic.

One more potential limitation of our experiments stems from the use of only one eye coil in each subject. Because we did not measure the position of both eyes, we cannot rule out the possibility that uncontrolled vergence had some impact on our findings. However, given our previous experience with measuring vergence using two eye coils (Uka & DeAngelis, 2003, 2006), we do not expect there to have been large errors in convergence.

### Causal inference and the detection of moving objects

To judge the motion of an independently moving object, a moving observer must first detect retinal motion that is inconsistent with the optic flow due to self-motion. This problem is one of causal inference (Kording et al., 2007; Shams & Beierholm, 2010; Shams, Ma, & Beierholm, 2005), in which the observer must decide whether an object's retinal motion was produced by self-motion alone or by the combination of self-motion and independent object motion. If the object's motion is judged as coming from a single cause (observer self-motion), the object is deemed part of the stationary world. If two causes are inferred (observer self-motion and object motion), the moving object should be segmented from the stationary background. This segmentation may occur in part automatically, as independently moving objects in the presence of optic flow appear to pop out to viewers (Rushton et al., 2007). Humans are more likely to detect independently moving objects that have larger speed mismatches with the inferred optic flow at the same location (Dokka, Park, Jansen, DeAngelis, & Angelaki, 2019; Royden & Connors, 2010; Royden, Parsons, & Travatello, 2016).

Failure to segment moving objects from optic flow can have a wide range of consequences, from missing a tennis volley to causing a car accident. Failure of segmentation can also impair the calculation of heading. If a moving object is judged as stationary relative to the world, its retinal motion will shift the focus of expansion of the optic flow field, biasing heading computations. Moving objects do tend to bias heading perception, especially if they obstruct the focus of expansion (Layton & Fajen, 2016b; Li et al., 2018; Royden & Hildreth, 1994, 1996; W. H. Warren & Saunders, 1995). However, biases in heading perception are smaller than they would be if the object's motion were treated as a world-stationary landmark generating optic flow (Raudies & Neumann, 2013). A causal inference model can account for biases in heading perception by predicting biases conditioned on whether an object is judged to be stationary (Dokka et al., 2019). If an independently moving object is incorrectly judged to be stationary, its motion will be integrated with the optic flow produced by self-motion, generating biases in perceived heading. If the object is inferred to be moving independently, it will be segmented from the optic flow field and its motion will have little or no effect on perceived heading. Thus, causal inference can explain biases in heading perception that are greatest when the discrepancy between object motion and optic flow is in an intermediate range (Dokka et al., 2019).

These considerations imply that the flow-parsing mechanism is not automatic and should be modulated by causal inference, such that background optic flow vectors should only be subtracted off when a subject believes that the object is moving independently. In contrast, when an object is believed to be stationary in the scene, its motion vectors should be spatially integrated with those of other background objects to improve estimates of self-motion. These predictions of causal inference are currently under study in our laboratories.

### Neurophysiological correlates of flow parsing

Because fore-aft self-motion generates nonuniform patterns of optic flow, flow parsing requires the subtraction of different vectors from different parts of the visual field. This subtraction likely relies on a neural substrate that represents object motion locally and in a maplike structure. Neurons in such an area would likely have relatively small, retinotopically organized receptive fields in order to discount the specific optic flow vectors passing over their receptive fields. One good candidate area for representing the effect of flow parsing on object direction is the middle temporal (MT) area, which has small receptive fields and a strong retinotopic organization (Albright, Desimone, & Gross, 1984; DeAngelis & Newsome, 1999; Van Essen, Maunsell, & Bixby, 1981; Zeki, 1974). Neurons in MT are strongly selective for motion direction (Albright, 1989; Albright et al., 1984; DeAngelis & Uka, 2003; Desimone & Ungerleider, 1986; Maunsell & Van Essen, 1983; Van Essen et al., 1981; Zeki, 1974) and speed (DeAngelis & Newsome, 1999; Liu & Newsome, 2003; Maunsell & Van Essen, 1983). MT activity reflects perceived motion direction (Britten, Newsome, Shadlen, Celebrini, & Movshon, 1996; Purushothaman & Bradley, 2005), even when the perceived motion does not correspond with retinal image motion (Krekelberg, Dannenberg, Hoffmann, Bremmer, & Ross, 2003; Luo et al., 2019; Rodman & Albright, 1989; Stoner & Albright, 1992). If MT represents the result of flow parsing, the observed biases in perceived object direction should correspond to a shift in MT's population response profile, when plotted as a function of direction preferences. This sort of shift could be generating by enhancing firing rates of MT neurons that prefer motion in the direction of the perceptual bias, while inhibiting neurons that prefer motion away from the direction of the bias.

Area MT is connected with the dorsal subdivision of the medial superior temporal area (MSTd), which is selective for heading from optic flow (Bradley, Maxwell, Andersen, Banks, & Shenoy, 1996; Duffy & Wurtz, 1995; Gu et al., 2006, 2008) and from vestibular cues (Bremmer, Kubischik, Pekel, Lappe, & Hoffmann, 1999; Duffy, 1998; Gu et al., 2006, 2007, 2008). A heading estimate from MSTd may be fed back to MT to modify its representation of object motion, thus mediating the perceptual compensation for self-motion. Several computational models of object motion perception during self-motion have been built around areas MT and MSTd (Layton & Fajen, 2016a, 2016b; Layton & Niehorster, 2019; Royden & Holloway, 2014), but the neural correlates of flow parsing are currently unknown. Having demonstrated that macaque monkeys exhibit perceptual biases characteristic of flow parsing, we are now well positioned to investigate the neural mechanisms of flow parsing.
